# Lenalidomide induced good clinical response in a patient with multiple relapsed and refractory Hodgkin's lymphoma

**DOI:** 10.1186/1756-8722-3-20

**Published:** 2010-05-28

**Authors:** Inga Mandac, Slobodanka Ostojic Kolonic

**Affiliations:** 1 Department of Internal Medicine, Merkur University Hospital, Zagreb, Croatia

## Abstract

**Background:**

A 24-year-old female patient was diagnosed with classic Hodgkin's lymphoma in clinical stage II, and combination chemotherapy followed by radiotherapy was initiated. During the following 5 years, the disease progressed despite several standard therapeutic approaches, including autologous and allogeneic stem cell transplantation.

**Methods:**

Lenalidomide (25 mg daily) treatment was then initiated in a continuous dosing schedule. Positron emission tomography scans were performed before and during lenalidomide treatment. Hematologic and laboratory values, as well as physical condition were also assessed before and during lenalidomide treatment.

**Results:**

Four months after continuous lenalidomide treatment, tumor load was significantly reduced, B symptoms had resolved, and the patient's physical condition had improved, allowing her to resume normal daily-living activities. Evaluations after 15 months of lenalidomide treatment indicated limited disease progression. Nevertheless, the patient was feeling well and maintaining a normal active life. Treatment was well tolerated, allowing the patient to remain on continuous dosing, which has now been maintained for 18 months.

**Conclusion:**

Daily, long-term lenalidomide treatment provided clinical benefit and was well tolerated in a patient with relapsed, advanced classic Hodgkin's lymphoma.

## To the editor

**Figure 1:**
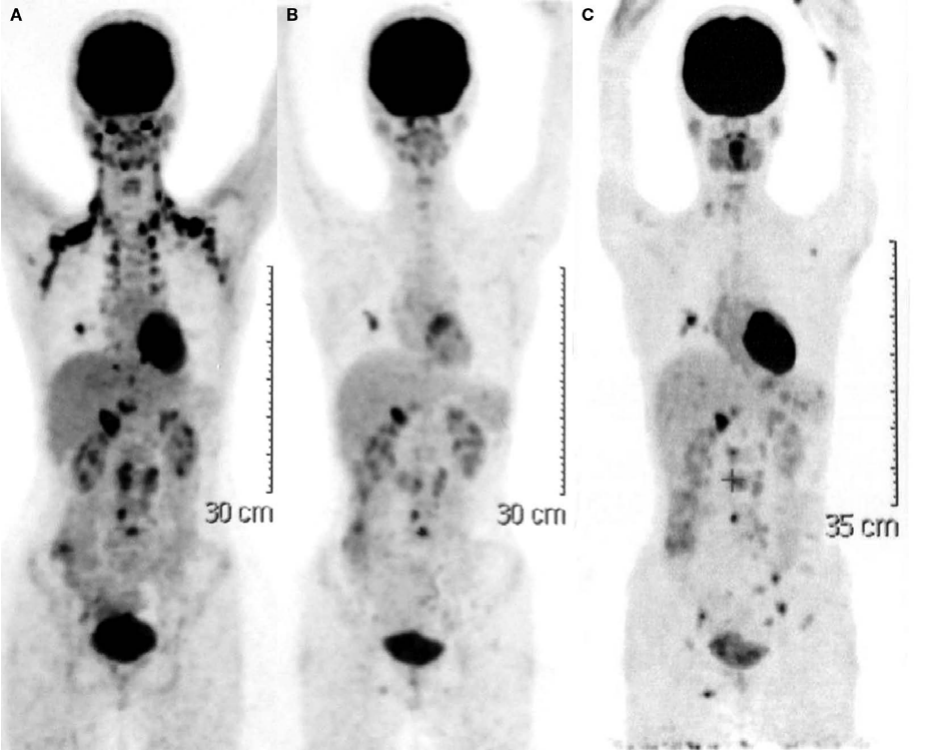
**Positron emission tomography scans**. (**A**) Before treatment with lenalidomide; (**B**) after 4 months of continuous lenalidomide treatment; and (**C**) after 15 months of continuous lenalidomide treatment.

In February 2003, a 24-year-old female was diagnosed with classic Hodgkin's lymphoma in clinical stage II. Her initial treatment consisted of 6 cycles of ABVD followed by involved-field radiotherapy of the mediastinal mass, and resulted in complete remission until November 2004, when she suffered a relapse. After a peripheral blood stem cell harvest, she underwent high-dose BEAM chemotherapy and an autologous peripheral blood stem cell transplantation (SCT), which was completed in January 2005 and resulted in complete clinical remission. In October 2005 a second relapse occurred, characterized by infiltration of the malignancy into lymph nodes in the L1 region and pelvis. The patient was treated with radiotherapy to the pelvis and 3 cycles of standard dose BEACOPP resulting in partial remission lasting until September 2006. In October 2006 a donor was identified and an allogeneic SCT was performed resulting in a complete response until February 2008 when positron emission tomography (PET) and multislice spiral computed tomography scans identified relapse (Figure 1A). Chemotherapy with one cycle of LVPP did not produce a clinical response, so in April and May 2008 the patient underwent two cycles of gemcitabine: 1,000 mg/m^2 ^on day 1 and day 8. A minor clinical response was observed but the patient's physical condition continued to worsen. Abdominal ultrasound revealed enlarged para-aortal, paracaval, mesenterial, and portal lymph nodes.

On June 25, 2008 treatment with continuous daily single-agent lenalidomide (25 mg) was initiated, outside of a clinical trial setting and based on discussions at the 10th International Conference on Malignant Lymphoma, Lugano, Switzerland, 2008. Prior to treatment initiation, the patient's hematologic and laboratory values were within or close to the normal range (Table 1). By October 2008, after 4 months of continuous lenalidomide, B symptoms had resolved, and she was able to tolerate treatment with no evidence of either hematologic or non-hematologic toxicity. The patient's physical condition improved and she was able to resume normal daily activities, such as her university studies. A PET scan performed 4 months after continuous lenalidomide (Figure 1B), revealed infiltrates in the middle lung lobe and in the right lobe, and the portal lymph node had shrunk. Metabolic activity was detected in the lungs and abdominal lymph nodes. The patient has remained on continuous lenalidomide since 2008. In a follow-up examination in August 2009 she was found to be in good physical condition and although her laboratory results showed mild leucopenia (World Health Organization hematologic toxicity scale grade 2), no dose reduction was required (Table 1). However, a third PET scan in September 2009 revealed new lesions in the lung, lymph nodes, and spleen, indicating disease progression (Figure 1C). Metabolic activity was detected in the lungs, left axillary lymph nodes, spleen, and vertebral column. Despite evidence of disease progression, the patient feels well, enjoys a good quality of life, is maintaining her university studies, and was able to go on vacation. She remains on continuous lenalidomide and her disease progression is expected to remain limited in the immediate future.

**Table 1:** Results of laboratory tests before initiation of lenalidomide treatment and after 12 months of continuous treatment.

Laboratory value	Reference range	Values before lenalidomide treatment	Values after 12 months of lenalidomide (25 mg/day) treatment
White cell count (× 10^9^/L)	3.4-9.7	4.31	2.2
Red cell count (× 10^12^/L)	3.86-5.08	4.52	3.9
Hemoglobin (g/L)	119-157	119	111
Hematocrit (L/L)	0.356-0.470	0.377	0.34
Platelet count (× 10^9^/L)	158-424	196	158
Mean corpuscular volume (fL)	83.0-97.2	83.4	83
Erythrocyte sedimentation rate (mm/3.6 ks)	4-24	26	26
Total protein (g/L)	66-81	70.5	67.2
C-reactive protein (mg/L)	< 5	12.1	Not done
Lactate dehydrogenase (U/L)	< 241	250	175
Aspartate aminotransferase (U/L)	8-30	20	16
Alanine aminotransferase (U/L)	10-36	6	16
Gamma-glutamyl transpeptidase (U/L)	9-35	15	10
Alkaline phosphatase (U/L)	54-119	147	124
Urea (mmol/L)	2.8-8.3	4.5	3.1 (blood urea)
Creatinine (qmol/L)	63-107	58	66

## Conclusions

The lenalidomide dose selected for this patient was based on the standard dose used in multiple myeloma, non-Hodgkin's lymphoma, and classic Hodgkin's lymphoma (25 mg daily for 21 days of every 28-day cycle) [1-4]. In this patient, no significant hematologic toxicity was detected with continuous lenalidomide, which has been ongoing for 18 months. The observed efficacy is consistent with data reported from two small studies [3,4]. Further clinical studies are needed to assess the clinical utility of lenalidomide in this indication, and to inform prescribing decisions on the most appropriate dose regimen.
